# Lower-limb surface electromyographic differences in roundhouse kicks between elite and sub-elite taekwondo athletes: a functional principal component analysis

**DOI:** 10.3389/fphys.2025.1690694

**Published:** 2025-10-24

**Authors:** Jianbo Sun, Yifei Wang, Jingyuan Sun, Delong Dong, Shazlin Shaharudin

**Affiliations:** ^1^ School of Health Sciences, Universiti Sains Malaysia, Kota Bharu, Malaysia; ^2^ College of Sports Sciences. Qufu Normal University, Jining, China; ^3^ College of Sports Sciences. Ludong University, Yantai, China

**Keywords:** integrated EMG, surface electromyography, functional principal component analysis, taekwondo, athletes

## Abstract

**Purpose:**

This study aimed to investigate the functional characteristics and distinctions in lower-limb electromyography (EMG) time-series data during roundhouse kicks performed by elite (n = 10) and sub-elite (n = 10) Taekwondo athletes, using Functional Principal Component Analysis (FPCA) to extract key parameters and identify critical phases of movement.

**Methods:**

EMG signals from 16 lower-limb muscles were collected via a Noraxon system synchronized with Vicon 3D motion capture. Group differences in integrated EMG (iEMG) were assessed using ANOVA, and muscles showing significant differences were further analyzed with FPCA (smoothing parameter e^−7^; eigenvalues >1; cumulative variance >85%).

**Results:**

Elite athletes demonstrated significantly higher iEMG values in specific muscles of both the supporting and kicking legs (p < 0.05). FPCA revealed higher scores for selected muscle components in the supporting and kicking legs among elite athletes (p < 0.05, ES = 0.64, *R* ^2^ = 92.6%; p < 0.05, ES = 0.66, *R* ^2^ = 88.8%; p < 0.01, ES = 0.53, *R* ^2^ = 94.8%). Notably, PC5 of the biceps femoris in the kicking leg was prominent during the kicking phase, while PC 4 of the gluteus maximus and biceps femoris in the supporting leg was critical during the recovery and end phases.

**Conclusion:**

These findings highlight muscle-specific contributions that differentiate kick quality between top-level and average athletes. FPCA offers a novel framework to assess movement quality, providing insights for technique improvement and supporting the development of automated performance evaluation systems in combat sports.

## 1 Introduction

For The roundhouse kick is one of the most representative techniques in Taekwondo competitions, as it combines speed, explosive power and rhythm control (Menescardi et al., 2019; Michael, 2020), while playing a central role in scoring and transitioning between offensive and defensive situations during actual combat (Falco et al., 2014; Fife et al., 2013). This technique requires athletes to execute a complete kinetic chain from preparation through force production to recovery within an extremely short time (Jung and Park, 2022). The quality of this action primarily depends on the coordinated force output from the lower limb muscles, particularly around the hip, knee and ankle joints (Thibordee and Prasartwuth, 2014). Muscular strength is a critical factor in leveraging the effectiveness of roundhouse kicks because it not only determines the kicking speed and impact force but also reflects an athlete’s technical proficiency and neuromuscular control.

Previous studies have utilized discrete parameters (peak muscular force and peak torque) to compare muscular performance among Taekwondo athletes with varying skill levels (Moreira et al., 2018; Moreira et al., 2016), and although these parameters reveal partial differences in technical proficiency (X. Wang, 2020),they fail to capture the dynamic changes in muscular output throughout the entire movement. In fact, muscular force variations during a roundhouse kick are continuous and linear (Górski et al., 2014), and traditional methods inadequately reflect the underlying temporal patterns inherent in these data. Functional Principal Component Analysis (FPCA) is an effective tool for capturing potential patterns and structural variations in time-series data (Tan et al., 2024). FPCA was first introduced into human biomechanics by Coffey in 2011 and has since attracted widespread attention from biomechanical researchers (Coffey et al., 2011). Warmenhoven conducted a case analysis comparing Australian international- and national-level rowing athletes using FPCA (Warmenhoven et al., 2019). Other researchers have explored the different functional characteristics and principal components in electromyography (EMG) signals in various phases of the gait cycle stab by applying FPCA to investigate differences in learning patterns between children with autism spectrum disorders and typically normal developing children, further examining FPCA’s performance with limited sample sizes through extensive simulations (Wang et al., 2025; Hasenstab et al., 2017). Thus, with FPCA’s gradual validation and expansion in various fields of sports science and bio-behavioral studies (Masiero et al., 2023), its advantage in managing high-dimensional and continuous biological signals has become increasingly apparent (Warmenhoven et al., 2021). Unlike traditional statistical methods that focus exclusively on discrete time points or maximal/minimal values, FPCA extracts dominant modes of variation from complete time-series data covering complex structural differences that are challenging to detect visually (Wang et al., 2016; Epifanio et al., 2008). This capability to model entire temporal cycles makes FPCA particularly suitable for processing typical functional data, such as muscular force curves, joint angle trajectories and electromyographic activity (Donà et al., 2009). FPCA decomposes time-series curves into principal components with clear physical interpretations (Donoghue et al., 2008), thereby revealing the force-output strategies employed by athletes of varying competitive levels during specific movement phases.

Therefore, applying FPCA to analyse lower limb muscular force curves when executing roundhouse kicks holds significant theoretical value and practical implications. On the one hand, it systematically identifies key variation patterns in muscular force output by capturing fundamental differences in temporal control and force production strategies among athletes of varying levels of skill. In contrast, principal component scores that serve as quantitative indicators of individual differences have the potential to become valuable tools for specialized training assessment and technique classification in future Taekwondo practice. This study extended the use of FPCA for analyzing muscular force outputs in kicking movements in order to address an underexplored area and contribute to the development of a muscular strength evaluation framework for elite and sub-elite athletes.

## 2 Methodology

### 2.1 Participants

This study had been approved by an institutional review board or ethics committee and appropriate informed consent was obtained (Supplementary Figure 1). G-Power software was used to calculate the sample size required for repeated measures of ANOVA, setting an effect size (f) of 0.3, significance level (α) of 0.05, statistical power of 0.95, involving six repeated measurements per participant. Each group comprised at least 10 participants, and recruitment occurred from November 5 to 5 December 2022. This study involved two distinct athlete-level datasets categorized as elite and sub-elite athletes. Table 1 shows information regarding the athletes included in this study. Athletes who participated in international or national competitions were classified as elite athletes, whereas those who competed at the provincial level in Shandong Province were classified as sub-elite athletes. All athletes were members of the Shandong Province Taekwondo Team and were recruited jointly by the team coach and the first author. Elite athletes were required to meet the following criteria, namely, ranked top three in the National Taekwondo Championships in the People’s Republic of China or within the top eight in international competitions, between 18 and 28 years of age, have at least 6 years of Taekwondo training experience as well as in good physical health and not dependent on any medication. Sub-elite athletes were required to meet the following criteria, namely, ranked top three in provincial Taekwondo competitions, between 18 and 26 years of age, have at least 2 years of Taekwondo training experience as well as in good physical health and not dependent on any medication. Elite athletes trained at least 25 h per week, whereas sub-elite athletes trained 10–15 h per week; all participants had no lower-limb injuries in the past 6 months.

**TABLE 1:** Summary of participants’ characteristics.

Group	Gender	Sample size	Age	Weight (kg)	Height (cm)	Training time (y)
Elite	Male	10	26.1 ± 2.3	72.6 ± 7.7	185.8 ± 8.6	7.8 ± 1.4
Sub-elite	Male	10	22.7 ± 2.7	70.8 ± 6.8	184.4 ± 9.7	4.2 ± 0.6

### 2.2 Measures

Surface electromyographic (sEMG) data were collected using a wireless Noraxon system (Noraxon United States Inc., Scottsdale, AZ) with a sampling rate of 2000 Hz. Based on previous research and the characteristics of this technique, EMG electrodes were placed on eight major lower-limb muscles according to SENIAM guidelines, such as the rectus femoris, biceps femoris, gluteus max-imus, vastus lateralis, tibialis anterior, gastrocnemius medialis, semitendinosus, and adductor longus (Sun et al., 2024). Simultaneously, 12 Vicon 3D motion capture system (Oxford Metrics Ltd., United Kingdom) cameras were used to record kinematic data at 200 Hz for phase synchronization.

### 2.3 EMG preprocessing and normalization

EMG was sampled at ≥1,000 Hz. Signals underwent DC offset and drift removal, band-pass filtering at 20–450 Hz (4th-order, zero-phase), and notch filtering at 50 Hz with harmonics when required. Data were full-wave rectified and smoothed to a linear envelope using a 4th-order, zero-phase low-pass filter at 15 Hz (within a 10–15 Hz range typical for ballistic tasks). Each waveform was time-normalized to 0%–100% of the kick cycle (preparatory stance to re-contact). Amplitude was normalized within subject and muscle to the maximum across the three valid trials.

### 2.4 Design and procedures

All the participants were required to wear tight-fitting pants on the day of the test. The testing procedure, movements and safety instructions were explained to them and basic physical measurements of the participants are recorded. Participants engaged in a brief 15-min warm-up session before the testing began that included dynamic stretching and specific preparation exercises to pre-vent sports injuries. The experiment was conducted at the Biomechanics Laboratory of Binzhou Medical University in Shandong, China, with all subjects being tested and guided by a laboratory staff member and the first author.

During the preparation process, one operator was responsible for attaching markers to the subject’s hip, knee and ankle joints, while another provided assistance. The body area where markers are placed should be shaved to remove any body hair, and the skin should be cleaned with alcohol to prepare the muscles for testing. After the alcohol has evaporated, the electrodes are positioned longitudinally (along the direction of the muscle fibres) on the muscle and secured in place using skin tape and adhesive materials for static recording in order to establish a model.

After the testing begins, participants follow verbal commands from the testing personnel. They execute the roundhouse kick technique to strike a specified target positioned at the height of each participant’s head. The study subjectively evaluated the participant’s kicking performance, and the test continued until six valid kicks were executed.

### 2.5 Roundhouse kick movement phases

**FIGURE 1:**
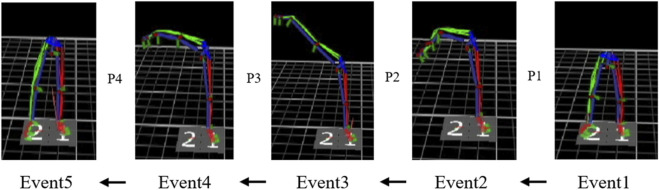
Phase division of the roundhouse kick.

In order to obtain a better understanding and analysis of the roundhouse kick technique, the movement was divided into four phases based on technical characteristics, such as initiation (P1), horizontal kick (P2), recovery (P3) and completion (P4) (Figure 1).

### 2.6 Statistical analysis

EMG data were processed using the MR3.16 software (Noraxon United States Inc., Scottsdale, AZ) to export integrated EMG (IEMG) values and activation sequences based on the equation below. EMG data were entered and organized using Excel 2018 (Microsoft Corp., Redmond, WA, United States). Differences in ΔIEMG between athletes of different skill levels were analysed using repeated measures of ANOVA in SPSS 29.0 (IBM Corp., Armonk, NY, United States), which yielded a significance level of α = 0.05. For iEMG ANOVAs, residual normality was assessed using the Shapiro–Wilk test and Q–Q plots, and homogeneity of variances via Levene’s test. Weighted coefficients (ω) were applied to calculate ΔIEMG and the Stepwise discriminant analysis was used to identify sensitive muscle indicators based on EMG score patterns. Once the sensitive indicators were selected, Cohen’s effect sizes (ES) were calculated and interpreted using thresholds suggested by Hopkins et al. (2009), Omnibus effects from repeated-measures ANOVAs are summarized by partial eta-squared with 95% CIs. For planned between-group comparisons (Elite vs. Sub-Elite) we report Hedges’ g with small-sample correction *J*, using the pooled-SD formula (or the *t*-based formula for Welch tests). For within-subject two-level contrasts we report Cohen’s d_z_ from the SD of paired differences. All effect sizes include 95% CIs and are interpreted as trivial (<0.20), small (0.20–0.59), moderate (0.60–1.19), large (1.20–1.99), very large (2.00–3.99), and extremely large (≥4.00). Raw mean differences (95% CI) are reported alongside standardized effects.

$$∆IEMG=\frac{\left(M_{iemg}+M_{iemg}\cdot \omega \right)-\left(M_{iemg}-M_{iemg}\cdot \omega \right)}{M_{iemg}+M_{iemg}\cdot \omega }$$

A functional data analysis was performed using the FDA FPCA toolbox in MATLAB (version 2024b). As EMG data from roundhouse kicks exhibit continuous data characteristics (Shang, 2014), the lower-limb surface EMG time series was fitted using a third-order Fourier basis with a smoothing parameter set at e−7 (Warmenhoven et al., 2021). The resulting functions were dimensionally reduced to principal components with cumulative variance contributions greater than 85% and eigenvalues above 1 (Ryan et al., 2006). For the primary analysis, we used the mean of six valid trials per subject. For sensitivity, trial-level PC scores were analyzed with a linear mixed-effects model including a random intercept for subject. We report ICC (3, 6) to characterize repeatability and the benefit of averaging (dos Santos Albarello et al., 2024).

The formula is as follows:

$$\int a\left(s,t\right)\;\xi \left(t\right)dt=\mu \;\xi \left(s\right)$$

$$\omega \left(t\right)=\xi \left(t\right)\times \sqrt{v}$$

$$Y_{i}=\int \xi \left(t\right)x_{i}\left(t\right)dt$$

## 3 Results

### 3.1 Comparison of lower limb muscular IEMG between different athlete levels

**FIGURE 2:**
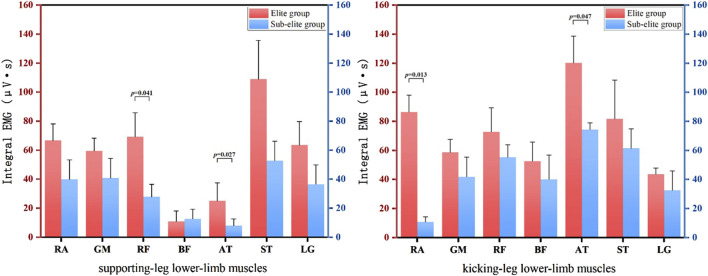
IEMG values of supporting leg and kicking leg for athletes of different levels.

This study analysed the kicking and supporting leg separately in order to compare the differences in IEMG between the two athlete groups. Figure 2 show the IEMG values during the kicking motion. The elite group displayed higher IEMG values compared to the sub-elite group. Each EMG waveform was time-normalized to 0%–100% of the roundhouse-kick cycle, defined from the preparatory stance (0%) to re-contact of the kicking foot with the floor (100%). Statistical analysis revealed significant differences (*p* < 0.05) in the rectus femoris (RF, *p* = 0.041) and anterior tibial (AT, *p* = 0.027) in the support leg, and rectus abdominis (RA, *p* = 0.013) and anterior tibial (AT, *p* = 0.047) in the kicking leg.

### 3.2 Functional principal component analysis of significant supporting leg muscles in iEMG time-series

**FIGURE 3:**
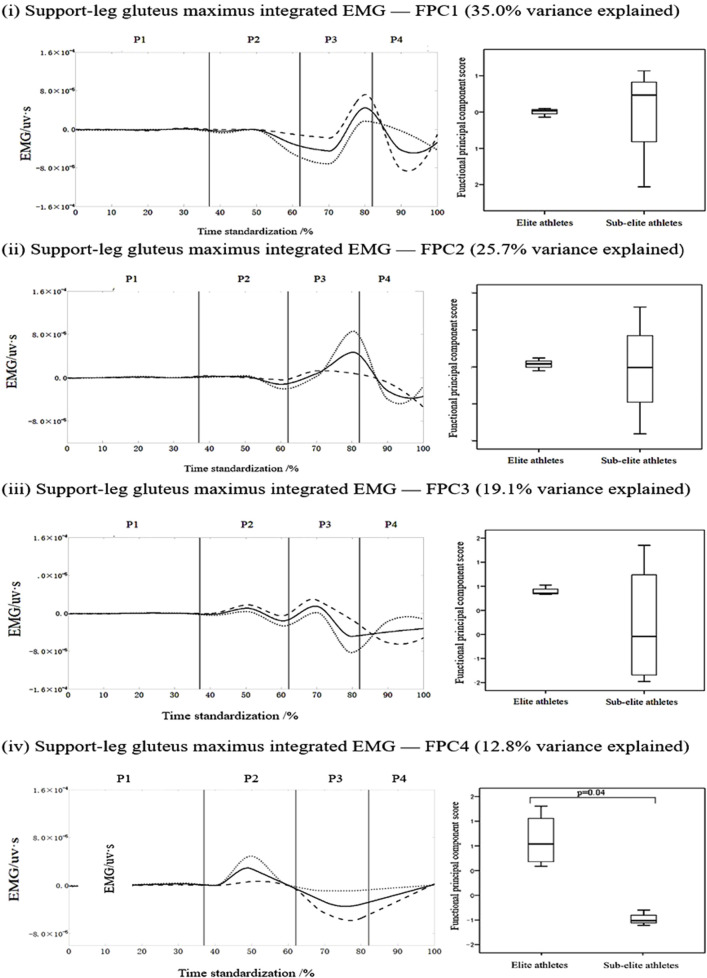
Mean ± loading coefficients and FPCA scores for the gluteus maximus in the supporting leg during roundhouse kicks in the two athlete levels.

Following the FPCA of the supporting-leg muscles across athletes of different performance levels, significant differences were identified in three muscles: the gluteus maximus, and biceps femoris. Figure 3 shows the FPCA results for the IEMG time-series curves of the Gluteus Maximus in the supporting leg during the roundhouse kick. After continuous registration and removal of the phase variance, the PC amplitude variation patterns were visualized. The solid line represents the mean IEMG curve, and the dotted and dashed lines represent the positive and negative deviations, respectively, based on the scaled PCs. The area between the curves indicates the location and extent of the variation. A positive PC score corresponds to a curve close to the dotted line, whereas a negative score corresponds to the dashed line.

Figure 3 shows the FPCA results for the IEMG time-series curve for the gluteus maximus in the supporting leg during the roundhouse kick. Data were reduced to four principal components with eigenvalues of 140.1, 102.6, 76.6, and 51.2, which explained 35%, 25.7%, 19.1%, and 12.8% of the total variance, respectively, with a cumulative variance of 92.6%. PC 1 showed amplitude variations around the first and second troughs as well as the first peak. PC 2 exhibited a variation pattern highly similar to that of PC 1. The variation in PC 3 ex-tended from the first trough to the end of the movement, whereas that of PC 4 varied be-tween the first peak and final trough. A statistically significant difference was found for PC 4 scores, with elite athletes scoring higher than sub-elite athletes (p = 0.04, Figure 3). For gluteus maximus (supporting leg), within-session repeatability across six kicks was moderate for averaging six trials (ICC (3,6) = 0.830, 95% CI 0.68–0.90; SEM = 0.045, CV = 5.56%).

**FIGURE 4:**
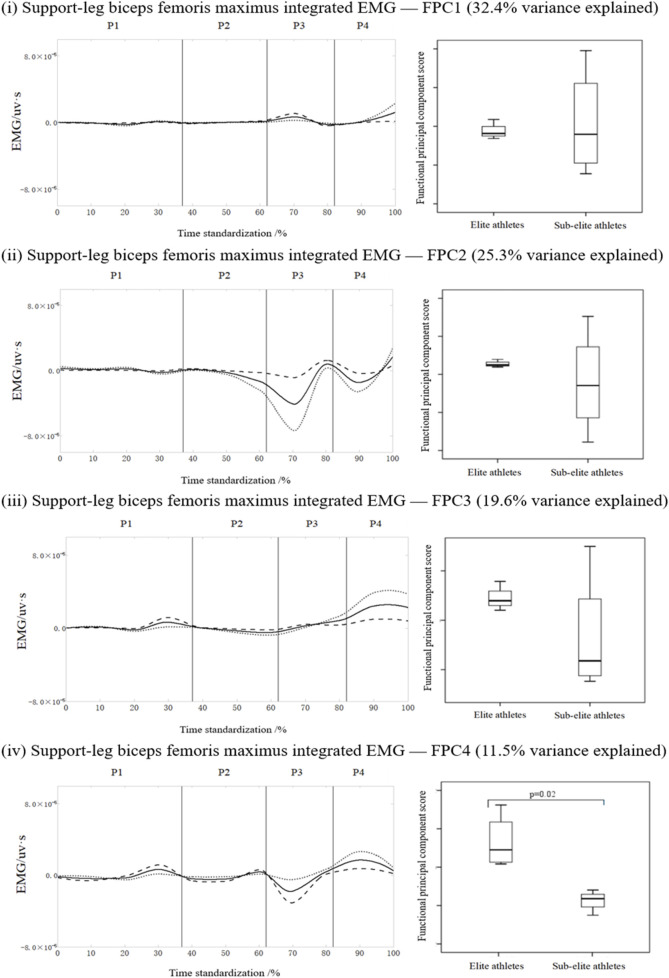
Mean ± loading coefficients and FPCA scores for the biceps femoris in the supporting leg during roundhouse kicks in the two athlete levels.

Figure 4 shows the FPCA results for the IEMG time-series curve for the biceps femoris muscle in the supporting leg. The curve was decomposed into four principal components with eigenvalues of 129.4, 101.3, 78.5, and 46.1, accounting for 32.4%, 25.3%, 19.6%, and 11.5% of the total variance, respectively, with a cumulative variance of 88.8%. PC 1 varied around the first trough and at the end of the motion, PC 2 varied around the first and second troughs, PC 3 focused on the first two peaks, and PC 4 varied around the initial peak, trough and second peak of the wave. No significant difference was observed for the first three PCs, but elite athletes scored significantly higher than sub-elite athletes, thus validating PC 4 (p = 0.02, Figure 4iv). For biceps femoris (supporting leg), within-session repeatability across six kicks was moderate for averaging six trials (ICC (3,6) = 0.82, 95% CI 0.69–0.90; SEM = 0.0337, CV = 5.8%).

### 3.3 Functional principal component analysis of significant kicking leg muscles in iEMG time-series

FPCA of all kicking-leg muscles revealed a significant difference in only one muscle, the biceps femoris, between elite and sub-elite athletes.

**FIGURE 5:**
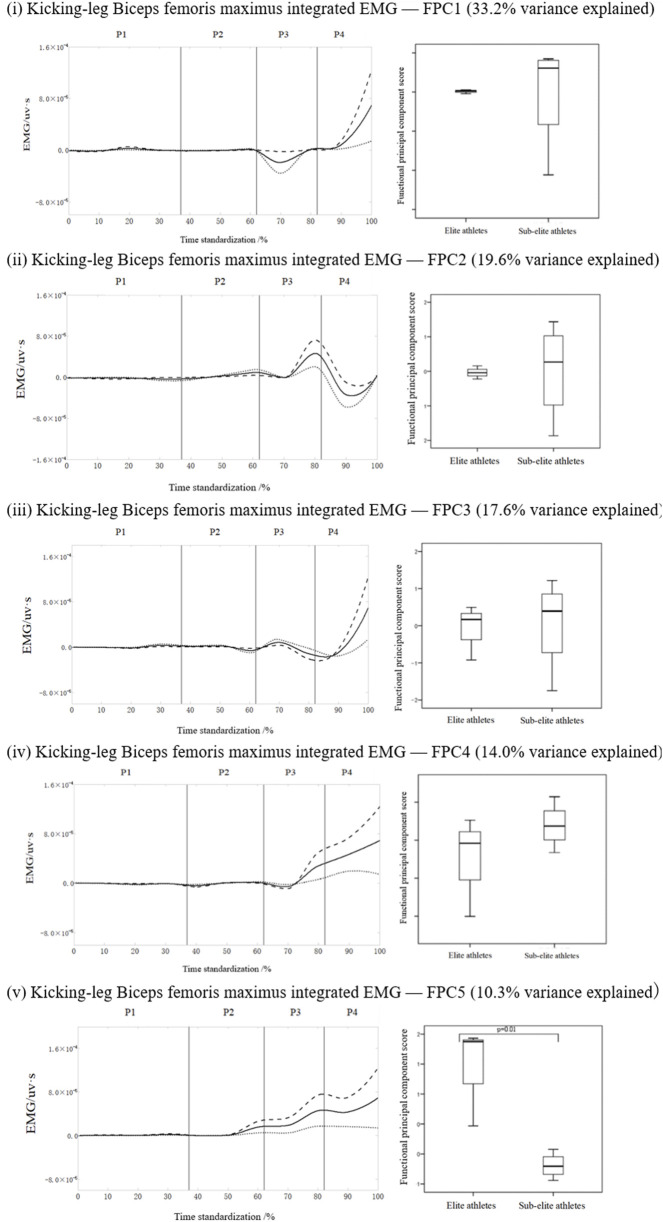
Mean ± loading coefficients and FPCA scores for the biceps femoris in the kicking leg during roundhouse kicks in the two athlete levels.

Figure 5 shows the FPCA results for the IEMG time-series curve for the biceps femoris in the kicking leg. The signal was reduced to five principal components with eigenvalues of 132.9, 78.3, 70.5, 56.1, and 41.3, corresponding to 33.2%, 19.6%, 17.6%, 14.0%, and 10.3% of the variance, respectively, with a cumulative variance of 94.8%. No significant difference was observed for the first four PCs; however, the elite group scored significantly higher than the sub-elite group in the fifth PCs (p = 0.01; Figure 5V). For biceps femoris (kicking leg), within-session repeatability across six kicks was moderate for averaging six trials (ICC (3,6) = 0.815, 95% CI 0.66–0.90; SEM = 0.0376, CV = 5.06%).

## 4 Discussion

Results of this study begin with conventional statistical comparisons of lower limb muscle activation when executing roundhouse kicks by elite and sub-elite Taekwondo athletes, revealing significant differences in the kicking and supporting legs. Subsequently, functional principal component analysis (FPCA) was applied to different muscles in order to explore the underlying associations between the skill level and EMG patterns. Principal components were extracted and presented as time-series curves by reducing the dimensionality of the EMG data in order to visualize the critical phases of muscle activation when executing the roundhouse kick. A dual-layer analytical strategy was employed with initial inferential statistics, followed by sensitivity mapping using FPCA, to describe the neuromuscular strategies of elite and sub-elite male Taekwondo athletes when executing the roundhouse kick. Direct comparison of integrated EMG (iEMG) showed that elite athletes had significantly greater activation in the rectus femoris and gastrocnemius muscles of the supporting leg, as well as the rectus abdominis and semitendinosus muscles in the kicking leg (p < 0.05, Cohen’s d = 0.70–1.03) compared to non-elite athletes. FPCA decomposed high-dimensional EMG time-series data into interpretable PCs, identifying four phase-sensitive discriminative fac-tors, such as PC4 of the gluteus maximus and biceps femoris in the supporting leg, and PC 5 and 4 of the biceps femoris and semitendinosus in the kicking leg. These PCs explained 62.1% of the total variance and corresponded to the late knee extension (P2) and leg recovery/landing (P3–P4) phases, thus, highlighting their biomechanical relevance. Elite athletes scored significantly higher in all four PCs, indicating superior amplitude modulation and temporal precision during the critical phases of kicking. Phase-specific sensitivity indices validated the findings of this study, in which the four discriminative muscles exhibited the highest overall sensitivity coefficients during the P2–P3 phase, whereas the sensitivity was uniformly low during the initiation phase (P1) in both groups.

This study identified four key functional principal PCs, namely, PC 4 in the gluteus maximus and biceps femoris in the supporting leg, and PC 5 and 4 in the biceps femoris and semitendinosus in the kicking leg. The amplitude variation patterns were consistent with the knee extension and leg retraction phases. Analysis of the iEMG data revealed the critical role of these muscles throughout the execution of the roundhouse kick, and this provided objective phase-specific activation profiles that can serve as quantitative references for high-level performances. Evaluation of the roundhouse kick performance was influenced by multiple factors, including athletic experience, physical conditioning and technical proficiency (Corcoran et al., 2024; Sant’Ana et al., 2017), all of which affect the intensity of muscle activation (Martone et al., 2024). The overall iEMG values clearly reflect the force-generating characteristics of different muscles involved in the kick (Wang and Zhao, 2023). Statistical analysis of iEMG revealed that elite athletes demonstrated significantly higher activation in the rectus femoris and gastrocnemius in the supporting leg and rectus abdominis and gastrocnemius in the kicking leg, in comparison to sub-elite athletes. Both groups also exhibited relatively higher activation of the semitendinosus and gluteus maximus in the supporting leg and gastrocnemius and semitendinosus in the kicking leg, suggesting the importance of these muscles during execution. These results align with previous research that emphasized the functional roles of the biceps femoris and semitendinosus (Ryan et al., 2006).

Kicking velocity places a high demand on the hip flexors and adductors, and a well-developed hip musculature contributes to greater force production when executing a kick. During the execution phase, the kicking leg transitions from knee elevation to hip rotation (Ema et al., 2017). At this point, the hip extensors replace the knee extensors as the dominant muscle group (Barnamehei et al., 2018; Sheng et al., 2016). Prior to this, the knee extensors primarily drive the motion, but the hip extensors then take over to propel the leg forward before the force generation shifts back to the knee extensors (Moreira et al., 2021). Data shows that the muscles involved in initiating the kick exhibit greater activation than those responsible for leg recovery. The support foot often undergoes subtle rotational adjustments as the whipping action develops (Thibordee and & Prasartwuth, 2014) in order to enhance power when executing the high-roundhouse kick. The primary function of the supporting leg is to maintain balance and facilitate trunk rotation and stability (Estevan et al., 2016). During this phase, the swinging leg performs a high knee lift to ensure the height of the kick. However, the activation of the ipsilateral rectus abdominis during the initiation phase is often overlooked. In contrast, this muscle exhibited significant recruitment during the kick initiation. Statistically significant differences were not observed in the erector spinae, gluteus max-imus, or semitendinosus–complex in the two groups. Notably, the electromyographic differences in the swinging leg in the two groups appeared to be smaller than those in the supporting leg. Therefore, coaches should emphasize eccentric loading exercises targeting the gluteus maximus and biceps femoris (Nordic hamstring curls and resisted leg retraction drills) to enhance kicking speed and reduce injury risk (Flaxman et al., 2021; Keerasomboon et al., 2024; Newham et al., 1983; Conneely et al., 2006; Keerasomboon et al., 2024).

This study explored the application of the FPCA for extracting the developmental characteristics of Taekwondo techniques, including parameter settings, interpretability of statistical indices and implementation conditions. The results demonstrate that FPCA is a promising tool for the quantitative identification and evaluation of technical performance in athletes. Future research should consider integrating machine learning classifiers, such as support vector machines, decision trees and neural networks, to identify and score technical movements in other sports disciplines (Fernando et al., 2024; Albertoni et al., 2023). This integration could contribute to the development of algorithm-based evaluation tools in the field of performance science. Moreover, increasing the sample size would allow for further exploration of the technical characteristics of world-class athletes.

Nevertheless, FPCA still has certain limitations. FPCA summarizes EMG time-series via eigenfunctions whose form depends on time-alignment, basis representation, and the smoothing penalty. Over- or under-smoothing can respectively attenuate transient bursts or amplify noise, and with modest samples the estimated eigenfunctions and the ordering of PCs may be unstable. Moreover, PCs are mathematical—not physiological—constructs: their sign is arbitrary, loadings may span multiple phases, and variance explained does not necessarily imply discriminative relevance. These properties constrain interpretability and inflate multiplicity when many muscles and PCs are tested. We mitigated these issues by standardizing preprocessing, selecting the smoothing parameter by cross-validation with sensitivity checks, using a pooled FPCA for between-group comparisons, limiting the number of retained PCs, controlling the false-discovery rate, and interpreting PCs through phase-specific loading patterns alongside effect sizes and confidence intervals.

## 5 Conclusion

Significant differences were observed in iEMG activity involving elite and sub-elite Taekwondo athletes when executing the roundhouse kick, especially in the rectus femoris and semitendinosus–semimembranosus complex muscles in the supporting leg and rectus abdominis and hamstrings in the kicking leg. Elite athletes exhibited greater activation and contribution from the tibialis anterior in the supporting leg and semitendinosus in the swinging leg than non-elite athletes. These findings suggest that sub-elite athletes may benefit from targeted neuromuscular training focusing on specific muscle groups (Jaskulski et al., 2024).

The FPCA identified several key phases and components critical for differentiating a kick. PC4 of the gluteus maximus and biceps femoris in the supporting leg and PC5 of the biceps femoris in the swinging leg were mainly identified as phase-sensitive indicators. The main between-group EMG variance occurred during the mid to late execution phases, namely, P2–P4 in the kicking leg and P2–P3 in the supporting leg, thus, indicating that the execution and recovery stages of the swing and support limbs are critical for distinguishing performance levels.

The FPCA results further demonstrate that neuromuscular distinctions between elite and sub-elite athletes are concentrated in the hip extensor coordination in the supporting leg and the deceleration control of the hamstrings in the swinging leg during the late execution and recovery phases. These insights provide actionable targets for evidence-based technique refinement and lay the foundation for automated performance assessment systems in combat sport biomechanics (Marinelli et al., 2024).

Recent work using skeleton-point tracking in taekwondo (Fernando et al., 2024) demonstrates that pose-estimation pipelines can quantify technique from video alone, offering non-contact data collection and high scalability for coaching or competition settings. These methods primarily describe external kinematics (segment/joint trajectories and spatiotemporal coordination). By contrast, our approach models internal neuromuscular control by applying FPCA to surface-EMG time series, yielding fPCs that summarize activation-shape features across the kick cycle. As such, skeleton-point and EMG-FPCA provide complementary information: the former reflects what the body does in space; the latter reflects how muscles are coordinated to produce it. We have added this comparison and roadmap to guide future development of an automated taekwondo performance evaluation system grounded in both movement kinematics and muscle activation. Looking ahead, these approaches can be integrated, and we anticipate the development of an automated taekwondo performance evaluation system grounded in kinematics and muscle activation.

## 6 Practical applications

The electromyographic (EMG) scoring and sensitivity analysis methods developed in this study offer a scientifically grounded approach for evaluating the quality of roundhouse kicks in Taekwondo athletes. These metrics serve as reliable indicators for distinguishing between high- and low-quality roundhouse kicks by quantitatively identifying which lower-limb muscles have the most influence on kick performance. Coaches and practitioners can utilize these findings to objectively assess kick execution, refine technical training and tailor strength programs toward the most sensitive and performance-relevant muscle groups. Furthermore, the EMG-based evaluation system can assist in athlete classification, injury prevention and the optimization of training loads by providing real-time feedback on muscle activation quality when carrying out kicking tasks.

## Data Availability

The original contributions presented in the study are included in the article/supplementary material, further inquiries can be directed to the corresponding author.
